# Short ROSE-Like RNA Thermometers Control IbpA Synthesis in *Pseudomonas* Species

**DOI:** 10.1371/journal.pone.0065168

**Published:** 2013-05-31

**Authors:** Stefanie S. Krajewski, Miriam Nagel, Franz Narberhaus

**Affiliations:** Microbial Biology, Ruhr University Bochum, Bochum, Germany; Max-Planck-Institute for Terrestrial Microbiology, Germany

## Abstract

The bacterial small heat shock protein IbpA protects client proteins from aggregation. Due to redundancy in the cellular chaperone network, deletion of the *ibpA* gene often leads to only a mild or no phenotypic defect. In this study, we show that a *Pseudomonas putida ibpA* deletion mutant has a severe growth defect under heat stress conditions and reduced survival during recovery revealing a critical role of IbpA in heat tolerance. Transcription of the *ibpA* gene depends on the alternative heat shock sigma factor σ^32^. Production of IbpA protein only at heat shock temperatures suggested additional translational control. We conducted a comprehensive structural and functional analysis of the 5′ untranslated regions of the *ibpA* genes from *P. putida* and *Pseudomonas aeruginosa*. Both contain a ROSE-type RNA thermometer that is substantially shorter and simpler than previously reported ROSE elements. Comprised of two hairpin structures only, they inhibit translation at low temperature and permit translation initiation after a temperature upshift. Both elements regulate reporter gene expression in *Escherichia coli* and ribosome binding *in vitro* in a temperature-dependent manner. Structure probing revealed local melting of the second hairpin whereas the first hairpin remained unaffected. High sequence and structure conservation of pseudomonal *ibpA* untranslated regions and their ability to confer thermoregulation *in vivo* suggest that short ROSE-like thermometers are commonly used to control IbpA synthesis in *Pseudomonas* species.

## Introduction

Pseudomonads are highly adaptive microorganisms with a high metabolic versatility that enables inhabitation of a wide variety of ecological niches [1]. The most prominent members of the *Pseudomonas* genus are the opportunistic human pathogen *P. aeruginosa* [2], the plant pathogen *P. syringae* [3] and *P. putida*, which has remarkable capabilities in bioremediation of organic pollutants including aromatic compounds [4]. Like all free-living microorganisms, pseudomonads are frequently exposed to changes in environmental conditions like variations in pH, osmolarity, nutrient availability and temperature. Temperature is one of the most important threats as a sudden upshift can cause severe damage of all cellular macromolecules in particular of proteins [5].

An universal protective response is the so-called heat shock response, evolved to avoid protein aggregation under heat conditions [5], [6]. Upon heat stress, a set of heat shock proteins (Hsp) are synthesized, which are mainly chaperones and proteases that either rescue misfolded proteins or promote their degradation [6], [7], [8]. In most Gram-negative bacteria, transcription of heat shock genes is controlled by the alternative sigma factor σ^32^ (RpoH) [9]. Under protein-denaturing conditions, the cellular σ^32^ level transiently increases due to a temporal stabilization and translational upregulation [6], [8], [10]. Homologues of *E. coli* σ^32^ are also found in several *Pseudomonas* species [11], [12].

At the translational level, heat shock gene expression can be regulated by RNA thermometers (RNATs). These elements are mRNA-inherent riboregulators responding to temperature changes [13]. Located in the 5′ untranslated region (5′UTR) of an mRNA, RNATs form secondary structures sequestering the ribosome binding site under low temperature conditions and thereby inhibiting translation initiation. With increasing temperature the secondary structure is destabilized and enables ribosome binding in order to initiate translation.

A moderately conserved class of RNATs are the ROSE-like elements (Repression Of heat Shock gene Expression) that control the synthesis of many small heat shock proteins (sHsp) and exhibit a complex secondary structure comprised of three to four hairpins [14], [15]. A major characteristic of ROSE-like RNATs is the U(U/C)GCU motif that blocks the SD sequence in the 3′ proximal hairpin by imperfect base pairing involving several non-canonical base pairs [14], [16], [17]. The best studied ROSE-like RNATs are the first described member ROSE_1_, regulating the *hspA* gene from *Bradyrhizobium japonicum*, and the *Escherichia coli ibpA* RNAT [16], [18], [19]. More than 40 candidates have been predicted upstream of the coding region of many bacterial small heat shock genes in diverse α- and γ-proteobacteria [14].

The *ibpA* genes of *P. putida* and *P. aeruginosa* are also preceded by ROSE-like RNATs [14]. The IbpA protein (inclusion body-associated protein A) belongs to the α-crystalline-type small heat shock proteins (sHsps) that bind to denatured and partly unfolded proteins under heat stress conditions [20]. Proteins bound to sHsps are maintained in a refolding-competent state and are thereby protected from aggregation [21].

In this study, we provide a comprehensive set of *in vitro* and *in vivo* experiments elucidating the molecular mechanism of *ibpA* regulation and the physiological role of the IbpA protein in representative *Pseudomonas* species.

## Experimental Procedures

### Bacterial growth conditions

Bacterial strains used in this study are listed in Table S1. *E. coli, P. putida and P. aeruginosa* strains were cultivated in LB medium and *P. syringae* in KB medium [22] at indicated temperatures. Media were supplemented with ampicillin (Ap, 150 µg/ml), kanamycin (Km, 50 µg/ml), tetracycline (Tc, 10 µg/ml) or rifampicin (Ra, 50 µg/ml) if required. For induction of the pBAD promoter in strains carrying translational reporter gene fusions, L-arabinose was added to a final concentration of 0.01% (w/v).

### Strain and vector constructions

Oligonucleotides and plasmids used in this study are summarized in Table S2 and S3. Recombinant DNA work was performed according to standard protocols [23]. The correct nucleotide sequences of all constructs were confirmed by automated sequencing (Eurofins, Martinsried, Germany).

For the construction of plasmid pBO500 (sequencing reaction; primer extension) a *P. putida ibpA* fragment ranging from −220 to +80 bp relative to translational start site was amplified (primer Pp_ibpA_PE_fw/Pp_ibpA_PE_rv) and cloned into EcoRI/HindIII sites of pUC18.

*P. putida ibpA* with promoter region (180 bp upstream and 150 bp downstream of the ATG) was amplified (primer Pp_ibpAprom_fw/Pp_ibpA+150_rv) and cloned into pUC18 via the primer derived EcoRI/HindIII restriction sites to obtain plasmid pBO1033.

To construct translational *bgaB* fusions the *ibpA* 5′UTRs were amplified by PCR and blunt-end subcloned into pUC18 (pBO504, pBO1046, pBO2954, pBO2955, pBO2956). The *bgaB* fusions were constructed by cloning via primer derived NheI/EcoRI sites into the corresponding sites of pBAD-*bgaB* (pBO1039, pBO1047, pBO2968, pBO2969 and pBO2967). Site-directed mutagenesis was performed on the pUC18 plasmids (Pp: pBO504 and Pp: pBO1046) with mutagenic primers (listed in Table S2) according to the instruction manual of the QuikChange® mutagenesis kit (Stratagene [now Agilent Technologies, Santa Clara, USA]). Mutated variants were afterwards transferred into the pBAD-*bgaB* plasmid as described above to obtain translational reporter gene fusions (Pp: pBO1040, pBO2982, pBO2976, pBO1044, pBO2983, pBO2984, pBO2977; Pa: pBO1505, pBO2979, pBO1508, pBO1507, pBO2987). For construction of the hairpin I deletion variants, the corresponding primers (Pp_ΔhpI_fw/rv; Pa_ΔhpI_fw/rv) were annealed, blunt-end subcloned into pUC18 (pBO1566, pBO1565) and transferred into the NheI/EcoRI sites of pBAD-*bgaB* (pBO2978, pBO2985).

Run-off plasmids used for *in vitro* transcription were constructed by blunt-end cloning of the 5′UTRs amplified with primers (Table S2) adding a T7 promoter sequence at the 5′ end and an EcoRV restriction site at 3′ end into SmaI site of pUC18. For structure probing the *ibpA* 5′UTRs plus one triplet coding region were cloned (pBO1513, pBO1515). Hairpin I deletion variants were constructed by amplification of the second hairpin and blunt-end cloning into pUC18 (pBO1569, pBO1571). For toeprinting experiments the 5′UTR and approx. 60 bp coding region were cloned into pUC18 (pBO1514, pBO1516). All point mutations were introduced by QuikChange® PCR (structure probing: pBO1562, pBO1556, pBO1557 and pBO1558; toeprinting: pBO1564, pBO1559, pBO1560 and pBO1561).

The *P. putida* Δ*ibpA* mutant strain was constructed as follows. The *ibpA* gene region with 150 bp upstream and downstream was amplified by PCR (primer Pp_ibpA+150_fw/Pp_ibpA+150_rv) and cloned into EcoRI/HindIII sites of pUC18 (pBO1031). A 32 nt fragment of the *ibpA* gene was deleted by restriction and re-ligation of the endogenous PstI restriction sites (pBO1032). The fragment was transferred into suicide vector pEX18Tc via the primer derived EcoRI/HindIII restriction sites (pBO1034). A kanamycin resistance cassette excised from plasmid pBSL14 was inserted into the PstI site of the *ibpA* fragment. The resulting plasmid (pBO1037) was introduced into *P. putida* PG5 by conjugation using the *E. coli* strain S17-1. Single cross-over integration mutants were selected on LB plates containing kanamycin and rifampicin. Single colonies were grown overnight in liquid LB without antibiotics and plated on LB containing kanamycin and 10% (w/v) sucrose to select for plasmid excision by double cross-over events. Kanamycin sensitive colonies were checked for *ibpA* deletion by colony PCR, Northern and Western analyses (data not shown).

### RNA preparation

Total RNA of cultured bacteria was isolated using the RNA preparation method described in [24] with minor modifications. 2.5 ml ice-cold stop buffer (100 mM Tris-HCl, pH 8, 200 mM ß-mercaptoethanol, 5 mM EDTA) was added to 5 ml bacterial culture. Cells were harvested and washed once with 1 ml cold washing buffer (10 mM tris-HCl, pH 8, 100 mM NaCl, 1 mM EDTA). Following steps of RNA isolation were performed as described. RNA concentrations were measured with a NanoDrop spectrophotometer ND-1000 (peQlab, Erlangen, Germany).

### Northern blot analysis

Northern blot analysis were performed as described previously [25] with the exception that DNA probes were used for transcript detection. Therefore a 393 bp fragment of the *P. putida ibpA* gene was amplified (primer Pp_ibpA_probe_fw/Pp_ibpA_probe_rv) and digoxigenin-labeled according to the instruction manual of the PCR DIG Probe Synthesis Kit (Roche, Basel, Switzerland). Quantification of band intensities was calculated with the Alpha Ease software (Alpha Innotech Corporation, San Leandro, USA).

### Primer extension analysis

Primer extension analysis was carried out as described before [26]. To map the *P. putida ibpA* transcriptional start site primer Pp_ibpA_PE_rv was used for reverse transcription on RNA isolated from bacteria that were heat-shocked for 5 min at 42°C. The DNA sequence reaction was performed with the same primer using the Thermo Sequenase cycle sequencing kit (USB, Cleveland, Ohio, USA) and plasmid pBO500 as template.

### 5′ Rapid amplification of cDNA ends (RACE)

5′ RACE experiments were conducted as described previously [27] with minor modifications. DNase treatment was performed with 2 U DNase I (Promega) and 40 U RiboLock (Fermentas [now Thermo Scientific, Waltham, USA]). Reverse transcription was carried out with SuperScript II (Invitrogen), according to the manufacturer's instructions. After PCR amplification with the adapterprimer and the gene-specific primer Pp_ibpA_RACE_rv, PCR products were separated on a 2% agarose gel. The prominent band was excised from gel, eluted (Omnipure-OLS® kit, OMNI life science, Bremen, Germany) and sent for sequencing with the gene-specific primer Pp_ibpA_RACE_rv (eurofins, Martinsried, Germany). A control PCR was performed on genomic DNA and RNA used for 5′ RACE.

### *In vitro* transcription of RNA

RNAs for structure probing and toeprint analyses were synthesized *in vitro* by run-off transcription with T7 RNA polymerase, from EcoRV-linearized plasmids (Table S3).

### Enzymatic and chemical RNA structure probing

Partial digestions of *in vitro* transcribed and [^32^P] 5′ end labeled RNA with ribonucleases T1 (Ambion, Austin, USA) and nuclease S1 (Fermentas [now Thermo Scientific, Waltham, USA]) were conducted according to [28], with the exception that 1 µl 5× reaction buffer for nuclease S1 (provided with nuclease) was used per reaction. Chemical structure probing with lead(II) and generation of alkaline ladder was conducted as described [18], [29].

### Toeprint analysis

Toeprint assays (Primer extension inhibition) were carried out as described [30], [31] with minor modifications. The [^32^P] 5′ labeled oligonucleotides Pp_runoff_Toe_rv (*P. putida*) and Pa_runoff_Toe_rv (*P. aeruginosa*) were used as primers for cDNA synthesis. *In vitro* transcribed RNAs annealed to the oligonucleotide were incubated at 25°C and 42°C with and without *E. coli* ribosomal 30S subunits. Instead of MMLV-RT, 2.5 U of AMV reverse transcriptase was used per reaction (USB, Cleveland, USA) and reverse transcription was performed at 37°C. Toeprint signals were identified by comparison with sequences generated with the Thermo Sequenase cycle sequencing kit (USB, Cleveland, USA) using plasmids pBO1514 and pBO1516 as templates and the same 5′ end labeled primers.

### Preparation of protein extracts and Western analysis (immunodetection)

According to their optical density (100 µl for OD_600_ = 1), cell pellets were resuspended in protein sample buffer (final concentration of 2% (w/v) SDS, 0.1% (w/v) bromophenol blue, 10% glycerol, 50 mM Tris/HCl, pH 6.8) and incubated at 95°C for 5 min. After centrifugation (1 min, 13 000 rpm) the protein extracts were subjected to SDS gel-electrophoresis (15% SDS PAA gels) and Western transfer using standard protocols [23]. *P. putida ibpA* was detected using a polyclonal antiserum against *A. tumefaciens* HspL [32] as primary antibody in a 1∶10 000 dilution. Goat anti-rabbit immunoglobulin G(H+L)-HRP conjugate (Bio-Rad, Munich, Germany) used in a 1∶3 000 dilution served as secondary antibody. Chemiluminescence signals were visualized using ECL Western blotting detection reagents (Amersham [now GE Healthcare, Munich, Germany]) and a chemiluminescence detector (FluorChem SP, Alpha Innotech, Biozym, Hessisch Oldendorf, Germany).

### β-galactosidase activity assay

*E. coli* DH5α cells harboring the *bgaB* plasmids were grown overnight in 10 ml LB at 25°C. 25 ml LB with ampicillin were pre-warmed to 25°C and inoculated with 2 ml overnight culture (OD_600_∼0.05). After growth to an optical density (OD_600_) of 0.5, transcription was induced with 0.01% L-arabinose (w/v) and 10 ml culture were shifted to pre-warmed 100 ml flasks at 42°C. After 30 min 400 µl samples were taken and used for galactosidase assays as described before [30].

### Bioinformatic tools/*in silico* methods

Sequence alignments were generated by the *ClustalW2* software obtained from http://www.ebi.ac.uk/Tools/msa/clustalw2/ [33], [34]. Secondary structure and consensus structure predictions were performed with RNAfold and RNAalifold (http://rna.tbi.univie.ac.at/, [35], [36]). A *ClustalW2* calculated sequence alignment was used for consensus structure prediction.

## Results

### Physiological role of the *Pseudomonas putida* IbpA protein

**Figure 1:**
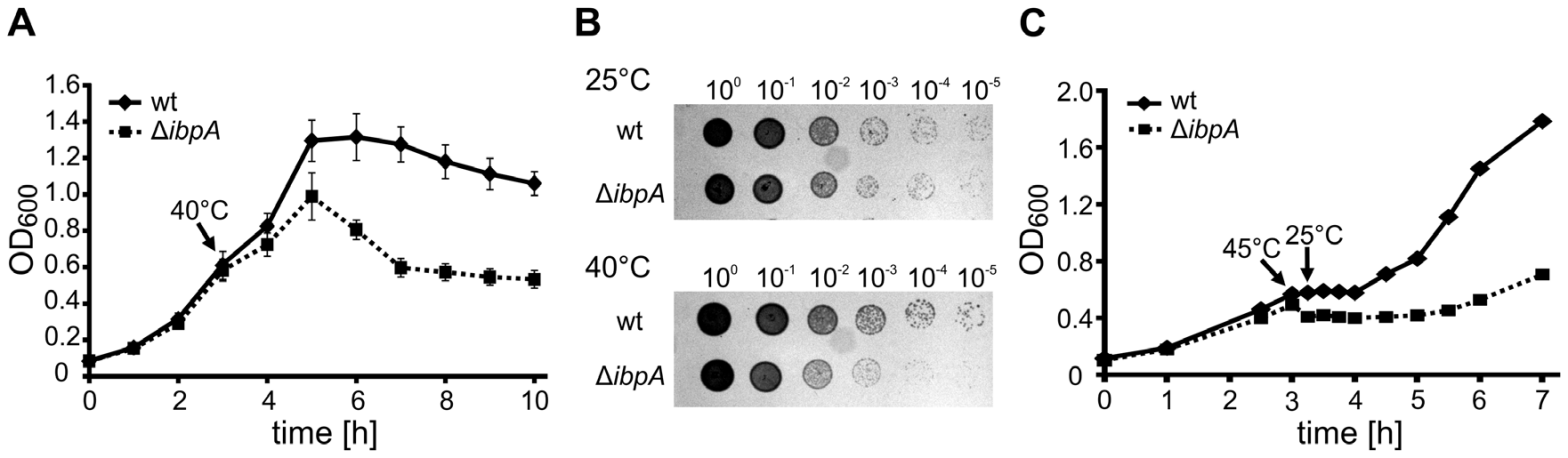
Phenotypes of *P.* *putida* wild type and Δ*ibpA* strains. A. Bacterial growth under heat stress in liquid media. *P. putida* wild type (wt) and Δ*ibpA* cells were grown to OD_600_ 0.5 at 25°C and then shifted to 40°C (black arrow). OD_600_ was monitored. Shown is the average result of three independent measurements with indicated standard deviations. B. Growth of *P. putida* wt and Δ*ibpA* at different temperatures on agar plates. Cells were grown to OD_600_ 0.5 at 25°C and serially diluted 10-fold, 3 µl of each suspension was spotted onto LB solid media and incubated at 25°C (upper) or 40°C (lower). C. Heat stress recovery of *P. putida* wt and Δ*ibpA*. Cells were grown to OD_600_ 0.5 at 25°C and heat-shocked for 15 minutes at 45°C (black arrow). Afterwards cells were incubated at 25°C to allow recovery. OD_600_ was monitored and plotted.

To gain insights into the cellular role of IbpA, we constructed a deletion mutant of the *P. putida ibpA* gene. The absence of IbpA was verified by colony PCR and Western blot analysis (data not shown). At 25, 30 and 37°C the growth of the *ibpA* mutant in liquid media was indistinguishable from that of the isogenic wild type (data not shown). However, a clear growth defect was observed when the temperature was raised to 40°C (Fig. 1A). Under this condition, the *ibpA* mutant grew slowly for additional two hours before the cell density dropped. Four hours after stress induction, the cell density of the mutant culture was approximately 60% lower compared to the wild type.

Consistent with the heat-sensitive phenotype observed in liquid cultures, a growth defect was apparent on solid media as well. At 25°C, the *ibpA* mutant exhibited wild type-like growth, as shown by equal amounts of living cells (Fig. 1B upper). In contrast, colony forming units (CFU) drastically decreased by several orders of magnitude in the Δ*ibpA* strain at 40°C compared to the wild type (Fig. 1B lower).

Furthermore, we compared the ability of the *P. putida* wild type and Δ*ibpA* strains to survive from transient exposure to a severe heat stress (Fig. 1C). After 15 min heat shock to 45°C and a subsequent shift to 25°C growth of the wild type ceased for approx. one hour before the cells recovered and started to grow normally. In contrast, the *ibpA* mutant exhibited a prolonged recovery phase demonstrating that IbpA is required for optimal recovery from exposure to high temperature. For approx. three hours, cell density of the Δ*ibpA* culture remained unchanged before the cells started to recover. These results clearly demonstrate the importance of the IbpA protein for the fitness of *P. putida* under heat stress conditions.

### Transcriptional control of *P. putida ibpA*

**Figure 2:**
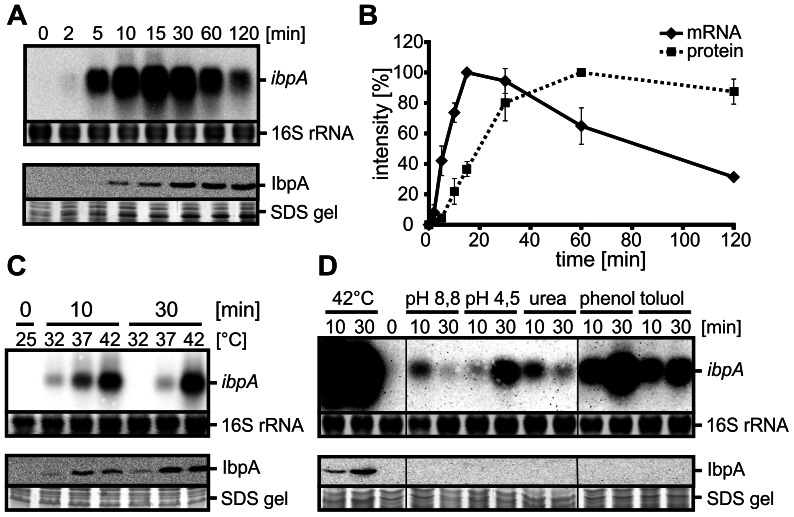
Transcriptional and translational regulation of the *P.* *putida ibpA*. A. Heat induction of *ibpA* transcript (444 nt) and IbpA protein (16.1 kDa). *P. putida* cells were grown to OD_600_ 0.5 at 25°C and heat-shocked at 42°C. Samples were taken at the indicated time points after the temperature upshift and probed by Northern and Western blot analyses. Upper panel: Northern blot and ethidiumbromide-stained 16S rRNA as loading control; lower panel: Western blot and corresponding section of the SDS gel. B. Quantification of *ibpA* transcript and IbpA protein amounts. Signal intensities were quantified with the Alpha Ease software, normalized to the strongest signals and plotted (*ibpA* mRNA: solid line, IbpA protein: dashed line). C. *ibpA* transcript and IbpA protein amounts at different heat stress scenarios. *P. putida* cultures were grown to OD_600_ 0.5 at 25°C and split to 32, 37 and 42°C. Samples were harvested 0, 10 and 30 min after temperature shift and analyzed via Northern and Western analyses as in 2A. D. Influence of various stresses on *ibpA* expression and IbpA protein synthesis. *P. putida* cells were grown to OD_600_ 0.5 at 25°C and exposed to following stress conditions: heat (42°C), osmotic stress (0.8 M urea), acidic stress (∼pH 4.5 with HCl), alkaline stress (∼pH 8.8 with NaOH), phenol (15 mM) and toluol (12 mM). Samples for Northern and Western analyses were taken 10 and 30 minutes after stress induction and analyzed as in 2A.

The physiological relevance of the *P. putida* IbpA protein prompted us to examine the expression profile of the *ibpA* gene. First we analyzed the time course of temperature-dependent *ibpA* expression and IbpA protein accumulation. *P. putida* cells were grown to exponential phase at 25°C and heat shocked to 42°C. Northern blot analysis using a specific DNA probe revealed strong heat induction of the *ibpA* mRNA (Fig. 2A). Maximal levels of the monocistronic *ibpA* transcript were detected 15 min after heat induction (Fig. 2B). In parallel, protein synthesis was examined by Western blot analysis using an *Agrobacterium tumefaciens* HspL antibody that cross-reacts with *P. putida* IbpA (Fig. 2A). Highest IbpA amounts were reached about half an hour later than maximal *ibpA* mRNA levels (Fig. 2B). Significant transcript and protein levels were detectable even 120 min after heat stress induction.

Next, the effect of heat stress intensity was investigated. *P. putida* cells grown at 25°C were exposed to 32, 37 and 42°C. Northern blot analysis revealed a gradual increase of *ibpA* transcript with increasing temperatures (Fig. 2C) whereas efficient translation into IbpA protein predominantly occurred at 37°C or higher temperature (Fig. 2C). Other stress conditions, such as low and high pH, urea or the addition of phenolic compounds, were also able to induce transcription of the *ibpA* gene (Fig. 2D). Interestingly, IbpA protein could exclusively be detected after a heat shock even though the *ibpA* mRNA was present under all tested stress conditions. This hints towards a posttranscriptional control mechanism acting downstream of transcriptional regulation through the heat shock sigma factor σ^32^.

**Figure 3:**
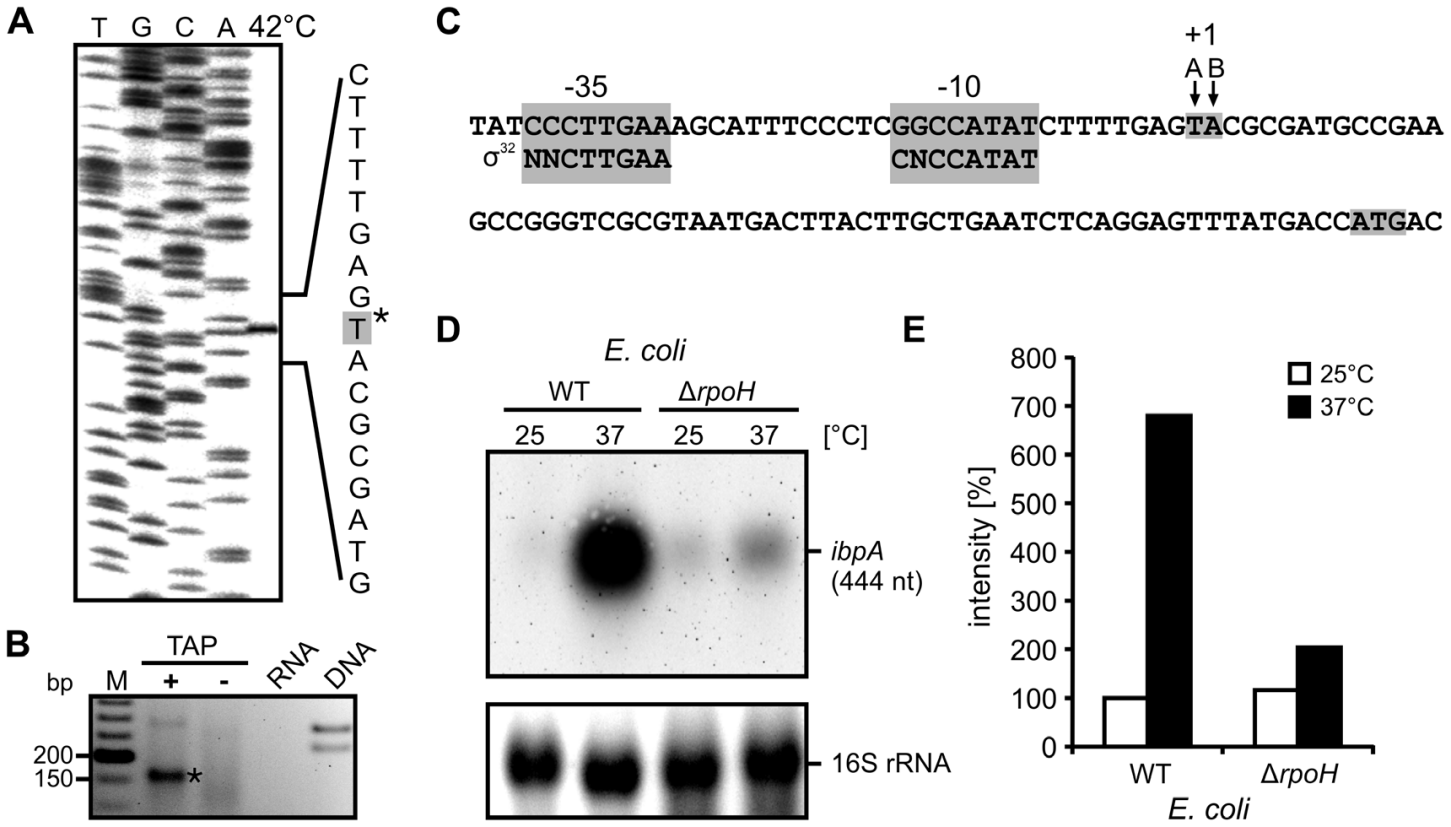
Determination of the *ibpA* transcriptional start site and verification of σ^32^-dependent transcriptional regulation. The 5′ end of the *P. putida ibpA* mRNA was mapped by primer extension (A) and 5′ RACE (B). A. Primer extension experiments were carried out with total RNA from *P. putida* cells heat-shocked to 42°C for 5 min. The corresponding DNA sequencing ladder (TGCA) was generated with the same end labeled oligonucleotide. 5′ ends of the mRNA are marked by an asterisk. B. 5′ RACE was performed with RNA extracted from *P. putida* cells after 10 min heat shock to 42°C. RNA was treated with (+) or without (−) tobacco acid pyrophosphatase (TAP) before adapter ligation and reverse transcription. An ethidiumbromide-stained agarose gel of 5′ RACE PCR products is shown. The fragment marked with an asterisk was eluted and sequenced. Control PCR was performed with total RNA used for the 5′ RACE and chromosomal DNA as template. C. Alignment of the *ibpA* promoter region with consensus sequence of *E. coli* σ^32^ promoter [37]. The −35 and −10 regions are shaded grey. The 5′ ends of the mRNA (+1) determined by primer extension (A) and 5′ RACE (B) are marked by arrows. D. Verification of σ^32^ (RpoH)-dependent regulation of *P. putida ibpA*. *E. coli* Δ*rpoH* and the isogenic wild type cells harboring a plasmid with the *P. putida ibpA* gene including its promoter region were grown to OD_600_ 0.5 at 25°C and heat-shocked at 37°C. Samples for RNA isolation were taken 10 min after stress induction and analyzed via Northern blot analyses with a *P. putida ibpA* specific DNA probe. E. Quantification of *ibpA* transcript amounts in *E. coli* wt and Δ*rpoH* at 25 and 37°C. Band intensities (D) were quantified using the Alpha Ease software and normalized to transcript detected in *E. coli* wt at 25°C.

To facilitate the reliable prediction of potential RNA structures involved in translational control, the 5′ end of the *P. putida ibpA* transcript was identified using primer extension and 5′ RACE (Rapid amplification of cDNA ends) experiments (Fig. 3A and B). Since the highest amounts of *ibpA* transcript were detectable after heat shock, total RNA isolated from cells heat shocked at 42°C was used. The primer extension analysis revealed a strong signal, which could be assigned to a thymidine 62 nucleotides upstream of the adenosine of the AUG start codon (Fig. 3A and C). 5′ RACE analysis was performed on untreated or tobacco acid pyrophosphatase (TAP)-treated total RNA. TAP treatment enhances signals from primary transcripts as it is specific for triphosphorylated 5′ ends and generates monophosphorylated ends suitable for adapter ligation. 5′ RACE-PCR gave rise to a prominent band, which exclusively appeared after TAP-treatment (Fig. 3B). Sequencing revealed a 5′ end just one nucleotide downstream (adenosine at position 61 upstream of the start codon) of the T determined by primer extension (Fig. 3C). In all following experiments, the longer transcript as determined by primer extension was used.

The region upstream of the determined transcriptional start site (+1) exhibits high sequence similarity with the −10 and −35 consensus sequence (NNCTTGAA-N(13-18)-CNCCATAT) of an *E. coli* σ^32^-type promoter suggesting transcriptional control by σ^32^ (Fig. 3C) [37]. To verify σ^32^-dependent regulation, a plasmid harboring the *P. putida ibpA* gene including its promoter region was constructed and transformed into *E. coli* Δ*rpoH* lacking σ^32^ and the parental *E. coli* wild type. In both strains only low amounts of the *P. putida ibpA* mRNA were detectable at 25°C (Fig. 3D). A sevenfold induction of *ibpA* expression was observed in the *E. coli* wild type cells after a temperature upshift to 37°C (Fig. 3E). In contrast, there was less than a twofold increase of *ibpA* transcript in the *E. coli* Δ*rpoH* strain (Fig. 3E). We conclude that transcriptional control of the *ibpA* gene depends on the heat shock sigma factor σ^32^.

### The *ibpA* 5′UTR confers temperature-dependent reporter gene activity

**Figure 4:**
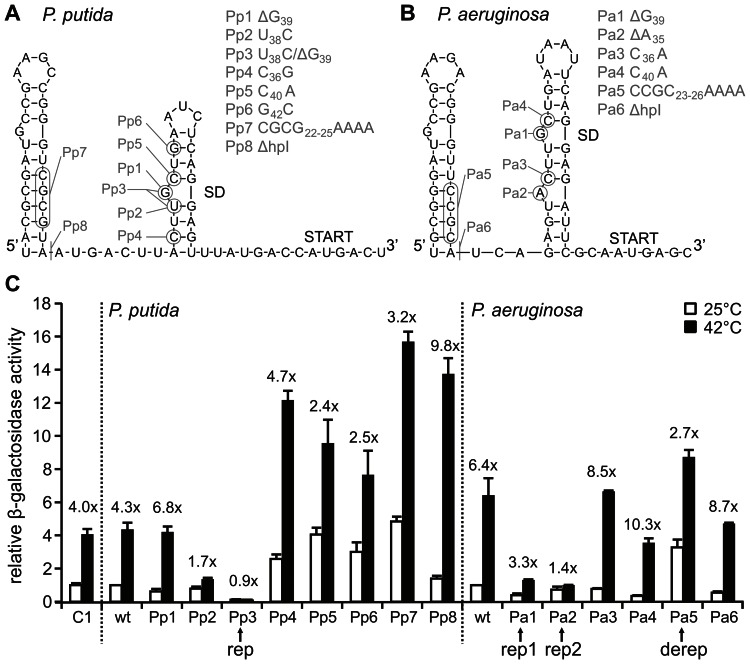
Translational control by the *P.* *putida* and *P. aeruginosa ibpA* 5′UTRs. A and B. Secondary structure prediction of the *P. putida ibpA* and *P. aeruginosa ibpA* 5′UTR calculated with RNAfold [36]. Introduced point mutations are indicated. Shine-Dalgarno regions (SD) and start codons are marked. C. Temperature-dependent expression of the *ibpA*-*bgaB* fusions. β-galactosidase assays were carried out with the *ibpA* wild type 5′UTRs and point-mutated variants translationally fused to *bgaB* in *E. coli* DH5α. Cells were grown at 25°C to OD_600_ 0.5, induced with 0.01% L-arabinose and shifted for 30 minutes to 42°C before β-galactosidase activity was measured. MU values for the *Pp ibpA* and the *Pa ibpA*-UTR (38 MU and 8 MU) at 25°C were set to 1. Activities from the mutated variants were normalized to the corresponding wild types. The average results of three independent measurements are shown with indicated standard deviations. An *E. coli ibpA*-*bgaB* fusion was used as a positive control (C1; 14 MU at 25°C) [18].

It has been postulated that translation of the *ibpA* genes of *P. putida* and *P. aeruginosa* is controlled by short ROSE-like RNA thermometers [14]. The transcriptional start of the *P. aeruginosa ibpA* gene was deduced from sequence comparison with the *P. putida ibpA* promoter region. *In silico* predictions of the *P. putida ibpA* 5′UTR (*Pp ibpA*-UTR; 62 nt) and the *P. aeruginosa ibpA* 5′UTR (*Pa ibpA*-UTR; 61 nt) suggested putative RNATs consisting of two hairpins (Fig. 4A and B). The SD sequences are located in the second, 3′ proximal hairpins and are imperfectly paired with the anti-SD sequence composed of the conserved U(U/C)GCU-motif typical for ROSE-like elements. The AUG start codons are predicted to be unpaired. The major difference between both structures is a more stable second hairpin in the *Pa ibpA*-UTR with three additional base pairs as compared to the *Pp ibpA*-UTR.

A well-established *E. coli* reporter gene system allows examination of temperature-mediated translational control by an RNAT [28]. In this system the UTRs are translationally fused to the *bgaB* gene encoding a heat stable β-galactosidase [38], and placed downstream of an L-arabinose-inducible promoter for temperature-independent control of transcription [39]. Fully consistent with the different thermodynamic stability of their second hairpin, the *Pp ibpA* and the *Pa ibpA*-UTR allowed basal β-galactosidase activity of 38 and 8 Miller units (MU), respectively. These values were set to 1, and β-galactosidase activity measured for the mutated variants were normalized to the corresponding wild types (wt; Fig. 4C). Comparable to the previously characterized *E. coli ibpA* RNAT (14 MU at 25°C; 4-fold induction), expression from the *Pp* and *Pa ibpA* 5′UTRs were induced 4.3 and 6.4 fold when cultures were shifted from 25°C to 42°C confirming the functionality of these RNATs. Again consistent with the higher stability of hairpin II, *Pa ibpA*-UTR did not reach reporter gene activity of the *Pp ibpA* constructs (51 versus 163 MU).

RNAT function depends on a delicate balance between destabilizing elements, such as mismatches and loops, and stabilizing elements like GC pairs to allow sufficient repression at low temperature and melting at higher temperature [40], [41]. Thus, single nucleotide exchanges aimed at disturbing this balance should result in deregulation of the temperature response. According to the secondary structure predictions (Fig. 4A and B), point mutations were introduced at different positions of the *Pp ibpA*-UTR (mutations Pp1-8) and *Pa ibpA*-UTR (mutations Pa1-8) and investigated for temperature-dependent translational control. First, point mutations intended to increase stability of the SD/anti-SD pairing (Pp1-3) were tested in the *Pp ibpA*-UTR. Deletion of G_39_ (Pp1) opposite the SD sequence resulted in slightly reduced expression level at 25°C and concomitant elevated heat induction (6.8 fold; Fig. 4C). In contrast, a loss of temperature-responsiveness (1.7 fold) occurred with mutation Pp2 (U_38_C) replacing a G-U by a more stable G-C pair. Enabling perfect pairing of SD and anti-SD by a combination of G_39_ deletion and U_38_C exchange (Pp3; henceforth called *Pp ibpA* rep for ‘repressed’) completely reduced β-galactosidase activity at low and high temperature, indicating a highly stable secondary structure incapable of melting. The second hairpin of the *Pp ibpA* 5′ UTR harbors three G-C pairs, one in the lower (C_36_-G_53_) and two in the upper stem (C_40_-G_50_ and G_42_-C_48_), which are considered to be important stabilizing elements of the *Pp ibpA* thermometer structure. Loosening of the lower G-C pair (C_36_-G_53_) by the mutation Pp4 (C_36_G) led to elevated expression at both temperatures. Mutations Pp5 (C_40_A) and Pp6 (G_42_C) each disrupting one of the upper G-C pairs also derepressed reporter gene activity and reduced temperature induction (2.4 and 2.5 fold). Destabilizing four G-C pairs in hairpin I (Pp7: CGCG_22–25_AAAA) resulted in increased reporter gene activity at 25°C and in reduced heat induction (3.2 fold).

Similar experiments were done in the *P. aeruginosa ibpA* 5′UTR possessing a more stable second hairpin. Expression was effectively repressed by deletion of single nucleotides, ΔG_39_ or ΔA_35_ (Pa1 and Pa2; 1.4 and 3.3 fold induction). In the remainder of this article, the variants ΔG_39_ or ΔA_35_ are referred to as *Pa ibpA* rep 1 and rep 2, respectively. Disruption of individual G-C pairs of the SD (Pa4: C_40_A, Pa3: C_36_A) showed wild type-like behavior, indicating that the second hairpin retained sufficient stability to inhibit translation at 25°C. As for the *Pp ibpA* UTR, a disruption of four G-C pairs belonging to the first hairpin (Pa5: CCGC_23–26_AAAA; henceforth called *Pa ibpA* derep for ‘derepressed’) resulted in an increased reporter gene activity at 25°C accompanied by reduced heat induction (2.7 fold).

To address the question whether the second hairpin is sufficient for RNAT function and to examine the importance of the first hairpin, variants consisting solely of the second hairpin (ΔhpI) were examined for temperature-dependent control. Low reporter gene activity was observed at 25°C for both variants (Pp8 and Pa6) demonstrating that effective inhibition had been preserved. Remarkably, a 9.8 and 8.7-fold increase of reporter gene activity was found for the *Pp* and *Pa* ΔhpI variants, respectively, when cultures were shifted from 25 to 42°C. In summary, the *P. putida* as well as the *P. aeruginosa ibpA* 5′UTRs exhibit short ROSE-like elements mediating temperature-dependent translational control *in vivo*.

### Binding of the 30S ribosomal subunit to the *ibpA* 5′ UTRs is temperature-dependent

**Figure 5:**
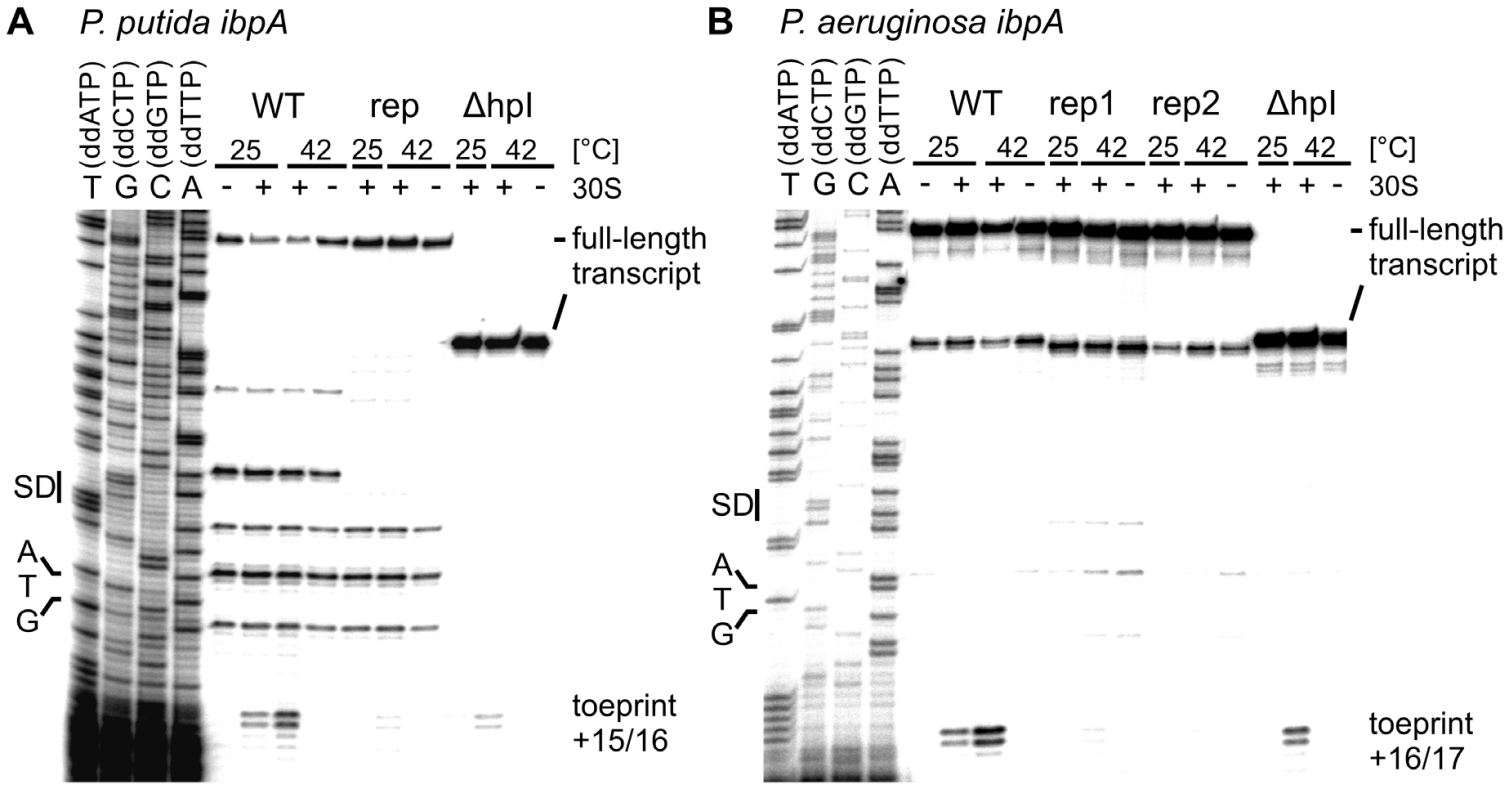
Temperature-dependent binding of the 30S ribosomal subunit to the *ibpA* 5′UTRs *in vitro*. Ribosome binding was shown by toeprinting experiments. The absence (−) or presence (+) of 30S subunits and incubation temperatures are indicated above the gels. Ribosome binding sites (SD) and the ATG start codons are marked to the left of the corresponding DNA sequencing ladder. Full-length products and terminated reverse transcription products (toeprints) relative to the A of the translational start codon are given on the right. A. Toeprinting analysis of *P. putida ibpA* wt, repressed (U_38_C/ΔG_39_) and Δhairpin I (ΔhpI) variants. B. Toeprinting analysis with *P. aeruginosa* wt, the repressed (rep1 ΔG_39_; rep2 ΔA_35_) and ΔhairpinI (ΔhpI) variants.

RNATs modulate the efficiency of translation initiation, which in turn correlates with the accessibility of the ribosome binding site [42]. Toeprinting (primer extension inhibition) analysis was performed to examine the binding of the 30S ribosomal subunit to the *ibpA* 5′UTRs (*Pp* wt and rep; *Pa* wt, rep1 and rep2). After annealing of a radio-labeled primer, *E. coli* 30S ribosomal subunit and the initiator tRNA^fMet^ were added to the *ibpA* 5′UTRs and incubated at 25 or 42°C. Efficient formation of translation initiation complexes gives rise to prematurely terminated products (toeprints) approximately 15 to 20 nucleotides downstream of the AUG start codon in primer extension reactions. At 25°C, only small amounts of toeprint product were obtained at position +15/+16 and +16/+17 for the *Pp* and *Pa ibpA* 5′UTR, respectively (Fig. 5A and B). The toeprint products increased at 42°C while full-length products decreased, both indicative of enhanced ribosome binding. For *P. putida* an approx. 4-fold and for the *P. aeruginosa* an approx. 3-fold increase in signal intensity was observed at high temperature as calculated by signal quantification. Apart from the fully extended and toeprint signals, truncated products occured in all lanes independent of ribosome addition. The presence of stabilizing point mutations (*Pp* rep; *Pa* rep1 and rep2) almost completely prevented ribosome binding at both temperatures (Fig. 5A and B). Additional toeprint experiments were performed with the *Pp ibpA* and *Pa ibpA* ΔhpI variants, containing the second hairpin only. Even these truncated variants prevented binding of the 30S ribosome to the SD sequences at 25°C and permitted formation of the translation initiation complex at 42°C. Taken together, the low efficiency of ternary complex formation at low temperature indicates that access of the ribosome to the *P. putida* and *P. aeruginosa ibpA* ribosome binding site is hindered by the thermometer structure. Moreover, the second hairpins alone seem to be sufficient for temperature-dependent inhibition of ribosome binding *in vitro* and *in vivo* (Fig. 4C).

### Heat-induced melting of the RNA thermometer

**Figure 6:**
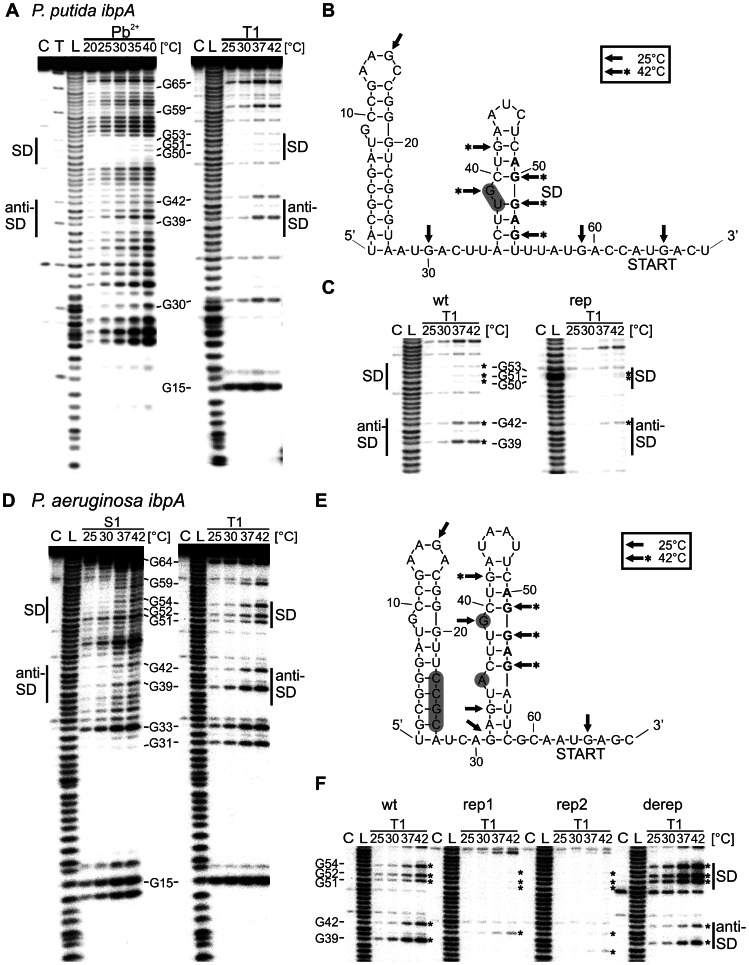
Secondary structure mapping and temperature-dependent alterations of the pseudomonal ROSE-like RNA thermometers. A. Structural analysis of the *P. putida ibpA* 5′UTR (wt) with lead (left) and RNase T1 (right). B. Structure model of the *P. putida ibpA* RNA thermometer with probing results. Cleavage sites introduced by RNase T1 at 25°C and 42°C are indicated by arrows and arrows with asterisks, respectively. The position of the stabilizing point mutation U_38_C/ΔG^39^ is shaded in gray. C. Comparative RNAse T1 probing of *P. putida* wt and the stabilized variant (rep U38C/ΔG39). Only the region of the gel corresponding to the SD and anti-SD region is shown. D. Structure probing of the *P. aeruginosa ibpA* 5′UTR (wt) with nuclease S1 (left) and RNase T1 (right). E. Structure model of the *P. aeruginosa ibpA* RNA thermometer with probing results. Cleavage sites introduced by RNase T1 at 25°C and 42°C are indicated by arrows and arrows with asteriks, respectively. Introduced point mutations of the stabilized (rep1 ΔG_39_; rep2 ΔA_35_) and destabilized (derep GGCG_23_-_26_AAAA) variants are shaded gray. F. Comparative RNA structure analysis of *P. aeruginosa* wt, rep1, rep2 und the derep variants. The gel region corresponding to the SD and anit-SD region is shown. Cleavage of 5′ end labeled RNAs were performed with nuclease S1 (0.25U), RNase T1 (0.002 U) or lead (20 mM). Reaction temperatures are indicated above the gels. Lanes C: control without enzyme or lead; lanes T: RNase T1 cleavage at 50°C; lanes L: alkaline ladder.

In order to determine the architecture and thermo-induced conformational alterations of the *P. putida* and *P. aeruginosa ibpA* RNATs we performed structure probing experiments at different temperatures. First, the secondary structure of the *P. putida ibpA* 5′UTR was mapped by enzymatic probing using RNases T1 (cuts 3′ of single-stranded guanines) and chemically with lead(II) (cleavage of single-stranded regions). The overall cleavage pattern (Fig. 6A) of the *P. putida ibpA* 5′UTR obtained with RNase T1 and lead(II) treatment at low temperature supports the predicted secondary structure (Fig. 6B). Lead(II)-induced cleavage around positions 46 confirmed the terminal loop region of hairpin I, whereas cleavage of G_15_ by RNase T1 in a temperature-independent manner conforms with the predicted loop region of hairpin II. Susceptibility of position 27–33 for lead(II) cleavage supports the single stranded region between both hairpin structures. Inversely, protection of positions 8–12 and 17–25 against RNase T1 and lead(II) cleavage confirmed the stem region in hairpin I (Fig. 6A). The double-stranded region of hairpin II was verified by the absence of lead(II) and RNase T1 cleavage at positions 38–45 and 49–53.

Increased susceptibility of the SD and anti-SD region to lead(II) cleavage with increasing temperatures provided evidence for temperature-dependent melting of hairpin II and liberation of the ribosome binding site (Fig. 6A, left). Cleavage of the SD and anti-SD region by RNase T1 exclusively at elevated temperatures supports the results from chemical structure probing (Fig. 6A, right and B). In contrast to these nucleotides that become accessible only after heat treatment, the single-stranded G_65_ of the start codon was cleaved by RNase T1 even at 25°C. A gradual increase of cleavage is most likely due to higher enzyme activity at higher temperature. Structure probing with RNase T1 in a temperature range from 20 to 50°C demonstrated melting of hairpin II whereas hairpin I remained unaffected (Fig. S1A). Perfect pairing of the SD in the rep variant led to a loss of reporter gene activity and prevented ribosome binding suggesting a secondary structure too stable for temperature-dependent melting (Fig. 4C and 5A). Impaired cleavage of RNase T1 in the SD sequence up to 42°C confirmed the high stability of hairpin II caused by the ΔG_39_/U_38_C mutation (Fig. 6C). The guanines of the SD sequence were protected against cleavage by RNase T1 even at 50°C (Fig. S1B).

Structure probing of the *P. aeruginosa ibpA* 5′UTR was performed with RNases T1 and nuclease S1 (cuts 3′ of unpaired nucleotides). Similarly to the *Pp ibpA* structure, the overall cleavage pattern was in good agreement with the predicted structure (Fig. 6D and E). As expected, S1 cuts around positions A_46_ and G_15_ confirmed the terminal loop regions of hairpin I and II. Conversely, protection from RNase cleavage (T1 and S1) at the region 8–13 and 17–30 supported formation of hairpin I, whereas absent S1 and T1 cleavage at the positions 35–43 and 53–58 verified base pairing in hairpin II. However, cleavage of G_31_ and G_33_ by RNase T1 and S1 but protection of U_56_, U_57_ and C_58_ might indicate transiently unpaired bases in the lower stem.

The G-C rich hairpin I was not temperature-responsive as it remained protected against cleavage by RNases T1 and S1 at 42°C. In contrast, hairpin II melted in a temperature-dependent manner as seen by RNase T1 and nuclease S1 cleavage (Fig. 6D and E). G_51_, G_52_ and G_54_ belonging to the SD region were marginally cleaved at 25°C indicating base pairing that prevents ribosome binding. At 42°C, the accessibility of the SD region for RNase T1 and S1 cleavage was significantly increased demonstrating melting of hairpin II (Fig. 6E). RNase T1 treatment performed up to 50°C showed further opening of hairpin II while hairpin I remained closed (Fig. S1B). The start codon, including G_64_, was susceptible to nuclease S1 and RNase T1 independent of the temperature.

Introduction of single nucleotide deletions potentially increasing the thermodynamic stability of hairpin II led to reduced expression accompanied by inhibition of ribosome binding (Fig. 4C and 5B). Structure probing of the corresponding *Pa* rep1 and rep2 RNAs at 42°C confirmed a thermo-stable hairpin II structure (Fig. 6F) protected from RNase T1 cleavage up to 45°C (Fig. S1B). Both stabilizing mutations had no impact on hairpin I conformation. In contrast, disruption of four G-C pairs belonging to hairpin I resulted in a derepression of thermometer function *in vivo* (Fig. 4C), which might be due to a thermo-labile secondary structure. In fact, the SD sequence forming guanines of the derep variant (CCGC_23–26_ AAAA) were accessible for RNase T1 cleavage even at 25°C (Fig. 6F). Moreover, destabilization of hairpin I affected the whole secondary structure (data not shown).

**Figure 7:**
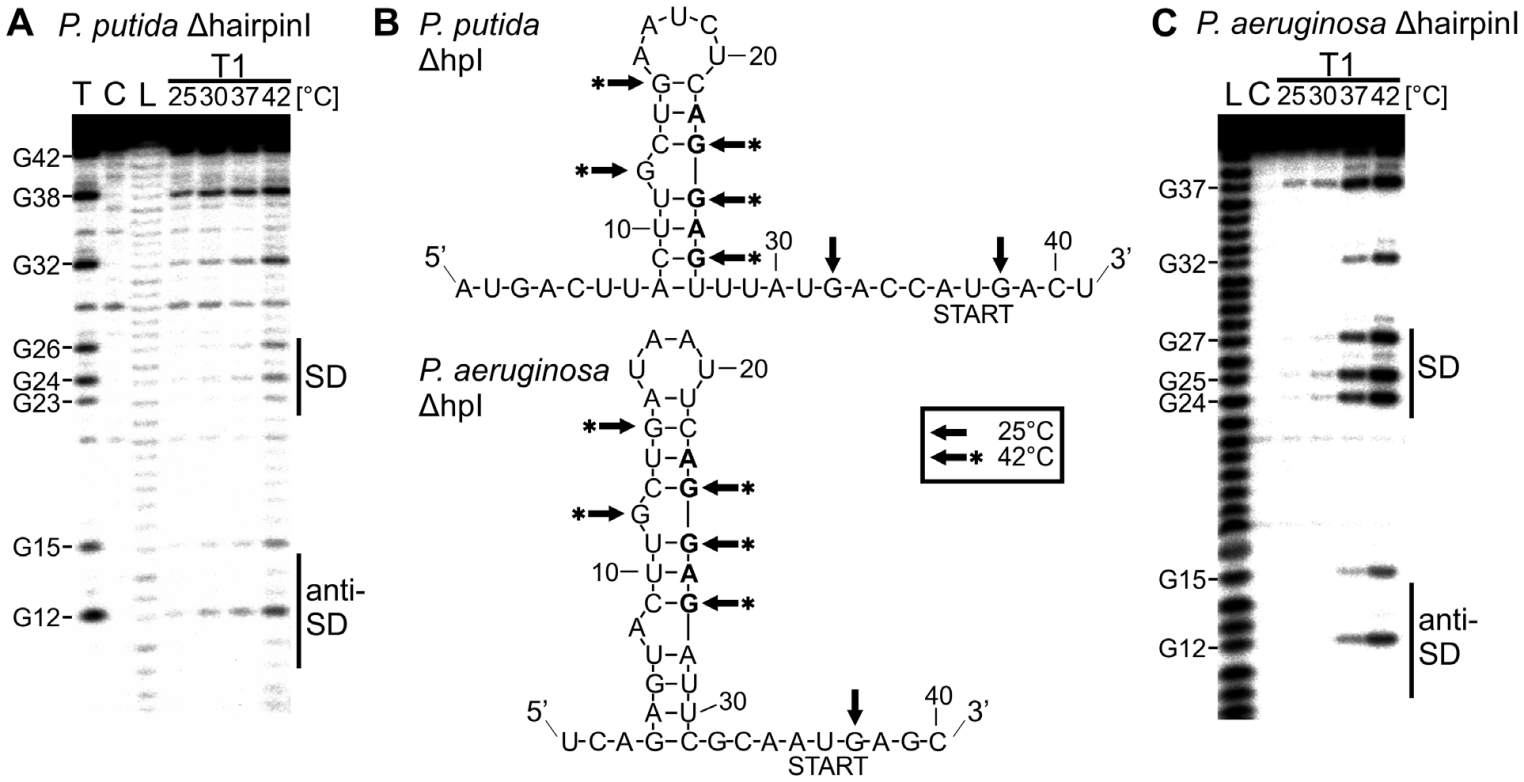
Structure probing of the second hairpin. Enzymatic structure probing of the isolated second hairpin of (A.) *P. putida* and (C.) *P. aeruginosa ibpA*. Cleavage of 5′ end labeled RNAs were performed with RNase T1 (0.002 U). Reaction temperatures are indicated above the gels. Lanes C: control without enzyme; lanes T: RNase T1 cleavage at 50°C; lanes L: alkaline ladder. B. Structure model of the *P. putida* (upper) and *P. aeruginosa* (lower) hairpinI deletion variants with probing results. Cleavage sites introduced by RNase T1 at 25°C and 42°C are indicated by arrows and arrows with asteriks, respectively. Nucleotides of the SD-region are depicted in bold letters and the AUG start codons are marked.

*In vitro* and *in vivo* experiments performed with constructs containing only the second hairpins (*Pp* und *Pa* ΔhpI) showed that they confer temperature-dependent regulation (Fig. 4C and 5). To determine whether the second hairpin forms a secondary structure, which blocks the SD sequence at low temperature, we performed structure probing of the ΔhpI variants of the *Pp* and the *Pa ibpA* RNATs using RNase T1 (Fig. 7 A and C). At 25°C, guanines of the SD sequences (*Pp*: G_23_, G_24_ and G_26_; *Pa*: G_24_, G_25_ and G_27_) were protected from RNase T1 cleavage indicating the formation of a hairpin structure. In contrast, the guanines of the start codon (*Pp*: G_38_; *Pa*: G_37_) were accessible to RNase T1 cleavage even at 25°C. With an increase in temperature, the SD guanines were liberated and cleaved demonstrating melting of the hairpin structure.

All these results provide strong evidence that the *ibpA* 5′UTRs of *P. putida* and *P. aeruginosa* fold into secondary structures at low temperature, which sequester the SD sequence and thereby reduce accessibility. A temperature increase results in melting of the dynamic second hairpin and liberation of the ribosome binding site. Furthermore, structural analysis point out the important role of hairpin II as thermo-sensing element.

### The ROSE-like RNAT is a conserved element controlling *ibpA* genes in divergent *Pseudomonas* species

**Figure 8:**
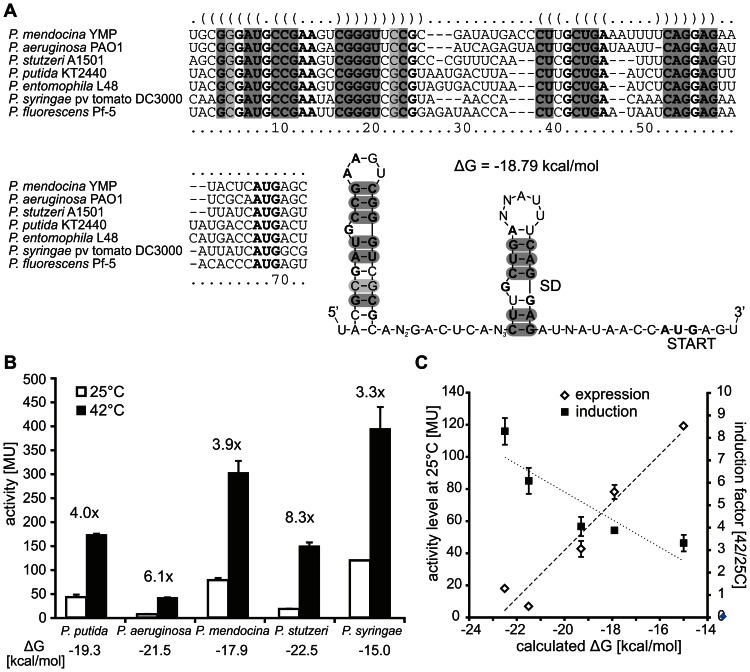
The *ibpA* RNA thermometer is conserved among *Pseudomonas* species. A. Sequence alignment and consensus structure of the *ibpA* 5′UTR of different *Pseudomonas* species calculated with *ClustalW2* and RNAalifold [33], [34], [36]. The calculated structure is given in dot-bracket annotation above the alignment. Sequence conservation is given in bold letters. Nucleotides highly conserved in structure are marked in grey (light grey: compensatory G-C pair in hairpinI). SD sequence and AUG start codon are marked in the consensus structure. N: no or any nucleotide. B. Comparative analysis of the thermoregulatory function of five pseudomonal *ibpA* 5′UTRs. β-galactosidase assays were carried out with *P. putida*, *P. aeruginosa*, *P. mendocina*, *P. stutzeri* and *P. syringae ibpA-bgaB* fusions in *E. coli* DH5α. Cells were grown at 25°C to OD_600_ 0.5, induced with 0.01% L-arabinose and shifted for 30 minutes to 42°C before β-galactosidase activity was measured. Shown is the average result of two independent measurements with standard deviations. Induction factors are shown above the fusions. Thermodynamic stability (ΔG) was calculated with RNAfold and is given below each RNAT [35], [36]. C. Influence of thermometer stability on expression level and induction factor. Reporter gene activity at 25°C (left y-axis) and induction factors (42/25°C; right y-axis) measured for the pseudomonal RNATs (B) are plotted versus their thermodynamic stability (calculated ΔG; x-axis). Dashed and dotted lines indicate the linear regression lines of the expression levels and induction factors, respectively.

As the *ibpA* 5′UTR of *P. putida* and *P. aeruginosa* are short functional RNATs, we examined the *ibpA* 5′UTRs of other *Pseudomonas* species for ROSE-like elements. Sequence comparison of the *ibpA* upstream regions from *P. putida, P. aeruginosa, P. syringae*, *P. stutzeri*, *P. mendocina*, *P. entomophila* and *P. fluorescens* displayed almost identical promoter-like sequences suggesting that these *ibpA* genes are all transcriptionally controlled by σ^32^ (data not shown). The 5′UTRs starting from the deduced transcriptional start sites were compared using the *ClustalW2* program prior to calculation of a consensus structure with the RNAalifold webserver [33], [34], [36]. The comparison revealed regions in the 5′UTRs that are highly conserved in sequence as well as in structure (Fig. 8A). The calculated consensus structure consists of two hairpins. The majority of the conserved nucleotides are involved in base-pairing of the stem regions, while loop-forming residues are more diverse. Hairpin I harbors seven highly conserved base pairs including a G-C base pair existing either in G-C or in C-G conformation (marked in light grey; Fig. 8A). Furthermore, all 5′UTRs exhibit the characteristic ROSE-motif (U(U/C)GCU) paired with the SD sequence in the second, 3′ proximal hairpin. Interestingly, the ROSE-motif/SD core region is flanked by G-C pairs in all compared sequences. To provide evidence for the conserved function of these ROSE-elements the *P. syringae*, *P. mendocina* and *P. stutzeri ibpA* 5′UTRs were analyzed in the *E. coli* reporter gene system. Consistent with the data presented in Fig. 4, induction factors of 4.0 and 6.1 were observed for the *P. putida* and *P. aeruginosa ibpA* 5′UTRs. At 25°C the *P. mendocina ibpA* fusion allowed a basal β-galactosidase activity of 78 MU that increased 3.9 fold to 303 MU after a shift to 42°C. Reporter gene expressed from the *P. stutzeri ibpA* 5′UTR increased from 18 MU approx. 8.3 fold to 149 MU when cultures were shifted from 25 to 42°C. The *P. syringae ibpA* fusion showed a higher basal ß-galactosidase activity of 215 MU at 25°C that increased 3.3 fold to 598 MU after a heat shock to 42°C (Fig. 8B).

In order to investigate the influence of the structural stability, the measured expression levels at 25°C (left y-axis) and induction rates (42/25°C; right y-axis) were plotted versus the calculated stability (x-axis), represented by the free energy levels of the RNAT structures (ΔG; Fig. 8C). Remarkably, there is a negative linear correlation between the structural stability and the expression level at 25°C and a positive correlation between structural stability and heat induction factor (activity 42/25°C). An increase in stability resulted in a decrease of reporter gene activity. For instance, the stable structure of the *P. aeruginosa ibpA* RNAT (ΔG −21.5 kcal/mol) led to a low basal activity of about 7 MU which increased 6.1 fold when cultures where shifted to 42°C. Contrary, the more unstable *P. mendocina ibpA* RNAT with a ΔG of −15 kcal/mol allowed an approx. 11 fold higher reporter gene activity at 25°C (78 MU) which in turn results in a reduced heat induction (3.9 fold).

Taken together, all five tested pseudomonal *ibpA* 5′UTRs are able to confer temperature-dependent translational control suggesting that short ROSE-like elements are a common regulatory feature in *Pseudomonas* species.

## Conclusion/Discussion

The moderately conserved ROSE-like elements are the most common class of RNATs known to date. Classical ROSE-type RNATs from *Rhizobium* species, *Caulobacter crescentus*, *Salmonella* and *E. coli* exhibit complex secondary structures comprised of three to four hairpin structures [14], [15], [19], [32]. Here, we provide comprehensive functional and structural studies showing that the *ibpA* gene is preceded by a short two-stem loop ROSE-like element in *Pseudomonas* species.

The 3′ proximal hairpin II harbors the SD sequence which is paired by the characteristic U(U/C)GCU-motif. In almost all investigated *Pseudomonas* species, this thermosensing hairpin is much shorter than the corresponding structure in all other known ROSE-elements [15], [18], [19]. Despite of its limited length, hairpin II alone is sufficient for thermoregulation *in vitro* and *in vivo*. Interestingly, the SD/anti-SD core region is flanked on both sites by G-C pairs. While loosening of the lower G-C pair in the shorter *P. putida* RNAT had a minor effect on translation efficiency, opening of the upper G-C pair resulted in loss of temperature-dependent repression. A crucial role of stabilizing G-C pair serving as molecular clamp was recently shown for the *Salmonella* fourU element [40]. Thus, it is conceivable that at least the upper G-C pair is an important stabilizing feature required for thermosensing by these short pseudomonal RNATs.

Beside the thermosensing hairpin II, the pseudomonal *ibpA* RNAT forms a hairpin I with a high content of G-C pairs unsusceptible to temperature-dependent structural alterations. The function of the 5′ proximal hairpins of ROSE-like RNATs is not fully understood. Deletion of the first three hairpins of ROSE_1_ from *B. japonicum*, the founding member of the ROSE family, resulted in reduced temperature responsiveness, indicating their importance for proper folding of the temperature-sensitive hairpin IV [16]. In contrast, hairpin I of the *Salmonella* fourU-type RNAT, consisting of two hairpin structures, is dispensable for RNAT function [28]. The *prfA* RNAT originating from *L. monocytogenes* consists of only one extended hairpin [43]. Recently, further RNATs comprised of a single short hairpin have been discovered in *Synechocystis* sp. PCC 6803 (*hsp17*), *Salmonella* and *E. coli* (*htrA*) [25], [44]. Nevertheless, high structural and sequence conservation in hairpin I of the pseudomonal *ibpA* RNATs suggests its importance for gene regulation. In support of this, we have not yet found any single-hairpin ROSE elements in sequence databases suggesting that the rather stable first hairpin is somehow beneficial to folding and/or stability of the loose thermosensory structure in a complex natural environment. In *E. coli*, a stem-loop structure located directly at the 5′ end of an mRNA was shown to stabilize the transcript [45]. Thus, hairpin I of the pseudomonal *ibpA* RNAT might prevent degradation of the transcript *in vivo* as reflected by the high abundance of the *ibpA* mRNA even two hours after heat shock induction. In support of this assumption, we found that the *ibpA* mRNA was more stable than other heat shock transcripts (*dnaK*, *groEL* or *grpE*) in the rifampicin-sensitive parental *P. putida* strain KT2440 (data not shown).

Different basal expression levels and induction rates of the *ibpA* RNATs selected for this study suggest that production of IbpA in *Pseudomonas* species has been adapted to the respective environmental niche. The interaction between the SD sequence of the mRNA and 30S ribosomal subunit is a key step in forming a translational initiation complex. As binding of the ribosomal subunit is basically a base-pair interaction between the SD sequence and the anti-SD sequence of the 3′ end of the 16S rRNA, it competes with the local intramolecular base-pairing of the mRNA [42]. Thus, efficiency of ribosome binding is primarily determined by the secondary structure around the translational initiation region and thus stability of mRNA structure correlates with translation efficiency [46], [47]. Moreover, the association and dissociation rates correlate with the structural stability of the mRNA [48]. mRNAs with instable secondary structures bound more tightly to the 30S ribosomal subunit [48]. Furthermore, a decrease of the minimum free energy (ΔG) by 1.4 kcal/mol equivalent to an increase in stability was shown to reduce translation efficiency by a factor of 10 [47]. The free energy of the thermosensing hairpins from various *Pseudomonas* species are in the range from −4.1 to −7.6 kcal/mol. Thus, their inhibitory effect on translation is in accordance with the hypothesis that stem-loop structures embedding the SD sequence affect translational initiation efficiency if their free energy is at least approx. −5 to −6 kcal/mol [49].

Consistent with these reports, comparison of the thermodynamic stability of the pseudomonal RNATs and their expression levels at low temperature revealed a clear negative linear correlation (Fig. 8C). At 25°C, RNATs with higher structural stabilities mediated lower reporter gene activity, i.e. better repression, than the more labile variants. For instance, the *P. aeruginosa ibpA* RNAT was a highly effective repressor element, whereas the *P. putida ibpA* RNAT permitted significant basal expression at low temperatures. A positive linear correlation was observed between RNA stability and heat induction (activity at 42/25°C). Apparently, efficient repression through stable structures can result in a higher relative induction potential when the temperature rises.

In many prokaryotes sHsps are dispensable [21]. For instance, an *E. coli ibpAB* mutant exhibits only a marginal temperature-sensitive growth defect that is more pronounced in cells additionally lacking the *dnaK* gene [50], [51]. In contrast, the *P. putida* IbpA protein is critical for fitness and survival under heat stress conditions and during the recovery phase. A similar observation was made in *Synechocystis*. Here, a mutant with a closed RNAT upstream of *hsp17* behaved like an *hsp17* deletion strain under stress conditions [44]. It had a clear growth defect and was compromised in photosynthetic activity. Intriguingly, a strain overproducing Hsp17 as a result of an open RNAT was protected during stress but delayed in recovery from stress conditions showing that the RNAT modulates chaperone production according to the cellular demand.

At least two mechanisms are responsible for proper expression of *ibpA*. The sigma factor σ^32^ couples *ibpA* transcription to the cellular protein folding status and thus integrates further input signals. Despite substantial RNA amounts, we did not detect IbpA after exposure to a variety of stresses suggesting that translational control dominates over transcriptional control. This is also true for the *Synechocystis hsp17* gene [44]. Translational control by a leaky RNAT could permit synthesis of a basal (not immunodetectable) level of IbpA protein able to assist in the multichaperone network even under non-heat stress conditions. In fact, IbpA synthesis in *P. putida* has been reported after exposure to aromatic compounds like toluene, *o*-xylene and 3-methylbenzoate and under filament-inducing conditions [52], [53].

In summary, the pseudomonal *ibpA* RNATs presented here are minimalistic ROSE-elements that contain a GC-rich first hairpin followed by a second temperature-responsive hairpin almost exclusively composed of the sensory core region flanked by two stabilizing G-C pairs. Recent global transcriptome analyses of *P. putida*, *P. aeruginosa* and *P. syringae* unraveled diverse riboregulators like small non-coding RNAs and riboswitches [54], [55], [56]. With our study, we add RNATs to the regulatory RNA inventory in *Pseudomonas* species.

## Supporting Information

Figure S1 **Fine-mapping of temperature-dependent melting of the pseudomonal RNA thermometers.**
(TIF)

Table S1 **Strains used in this study.**
(DOCX)

Table S2 **Oligonucleotides used in this study.**
(DOCX)

Table S3 **Plasmids used in this study.**
(DOCX)
