# Supplementary figures and images for: Short ROSE-Like RNA Thermometers Control IbpA Synthesis in Pseudomonas Species
Source: PLoS One. 2013 May 31;8(5):e65168. doi: 10.1371/journal.pone.0065168 (PMC3669281; doi:10.1371/journal.pone.0065168)

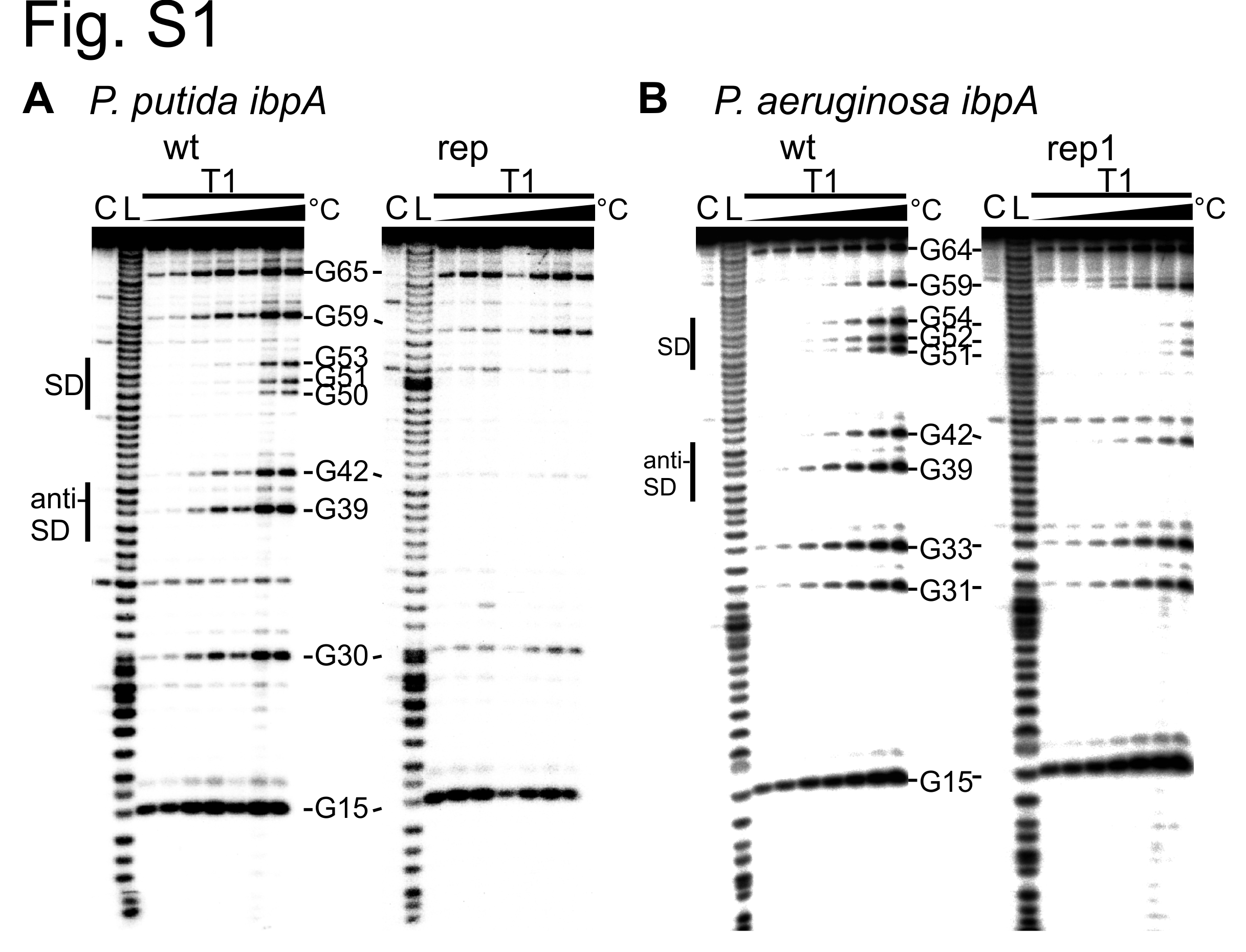

Supplement: Figure S1 — Fine-mapping of temperature-dependent melting of the pseudomonal RNA thermometers. (TIF) [file pone.0065168.s001.tif]
