# Supplementary material for: Short ROSE-Like RNA Thermometers Control IbpA Synthesis in Pseudomonas Species
Source: PLoS One. 2013 May 31;8(5):e65168. doi: 10.1371/journal.pone.0065168 (PMC3669281; doi:10.1371/journal.pone.0065168)
Supplement: Table S1 — Strains used in this study. (DOCX) [file pone.0065168.s002.docx]

**Supplementary table S1: Strains used in this study**

| Strain | Relevant characteristics | Reference or source |
| --- | --- | --- |
| ***Escherichia coli*** |  |  |
| DH5α | *supE44,* Δ*lacU169* (ψ80*lac*ZΔM15)*, hsdR17, recA1, gyrA96, thi1, relA1* | [[1](#_ENREF_1)] |
| S17-1 | Ec294::[RP4-2(Tc::Mu)(Km::Tn7), *pro*, *res*, *rec*A, Tp^R^ Sm^R^ | [[2](#_ENREF_2)] |
| MC4100 | F- *araD139* Δ(*argF*-*lac*)U169 *rspL150* *relA1 flb5301 fruA25 deoCl* *ptsF25 e14-* | [[3](#_ENREF_3)] |
| Δ*rpoH* | MC4100 Δ*rpoH30*::kan *zhg-50*::Tn10 [λpF13-(*groE*_p_-lacZ^+^)] | [[4](#_ENREF_4)] |
| ***Pseudomonas* strains** |  |  |
| *P. putida* PG5 | KT2440 Rif^r^ Nal^r^ (wild type) | [[5](#_ENREF_5)] |
| *P. putida* Δ*ibpA* | PG5 Δ*ibpA32*::[>km]^R^ | This study |
| *P. aeruginosa* PAO1 | Wild type | [[6](#_ENREF_6)] |
| *P. syringae* pv. *tomato* DC3000 | Wild type | [[7](#_ENREF_7)] |
| *P. mendocina* YMP | Wild type | [[8](#_ENREF_8)] |
| *P. stutzeri* A1501 | Wild type | [[9](#_ENREF_9)] |

**References**

1. Hanahan D (1983) Studies on transformation of *Escherichia coli* with plasmids. J Mol Biol 166: 557-580.

2. Simon R, Priefer U, Puhler A (1983) A Broad Host Range Mobilization System for *In Vivo* Genetic Engineering: Transposon Mutagenesis in Gram Negative Bacteria. Nat Biotech 1: 784-791.

3. Peters JE, Thate TE, Craig NL (2003) Definition of the *Escherichia coli* MC4100 genome by use of a DNA array. J Bacteriol 185: 2017-2021.

4. Zhou YN, Kusukawa N, Erickson JW, Gross CA, Yura T (1988) Isolation and characterization of *Escherichia coli* mutants that lack the heat shock sigma factor s^32^. J Bacteriol 170: 3640-3649.

5. Petruschka L, Burchhardt G, Muller C, Weihe C, Herrmann H (2001) The *cyo* operon of *Pseudomonas putida* is involved in carbon catabolite repression of phenol degradation. Mol Genet Genomics 266: 199-206.

6. Holloway BW (1955) Genetic recombination in *Pseudomonas aeruginosa*. J Gen Microbiol 13: 572-581.

7. Cuppels DA (1986) Generation and Characterization of Tn5 Insertion Mutations in *Pseudomonas syringae* pv. *tomato*. Appl Environ Microbiol 51: 323-327.

8. Hersman LE, Forsythe JH, Ticknor LO, Maurice PA (2001) Growth of *Pseudomonas mendocina* on Fe(III) (hydr)oxides. Appl Environ Microbiol 67: 4448-4453.

9. Yan Y, Yang J, Dou Y, Chen M, Ping S, et al. (2008) Nitrogen fixation island and rhizosphere competence traits in the genome of root-associated *Pseudomonas stutzeri* A1501. Proc Natl Acad Sci U S A 105: 7564-7569.
