# Supplementary material for: Short ROSE-Like RNA Thermometers Control IbpA Synthesis in Pseudomonas Species
Source: PLoS One. 2013 May 31;8(5):e65168. doi: 10.1371/journal.pone.0065168 (PMC3669281; doi:10.1371/journal.pone.0065168)
Supplement: Table S2 — Oligonucleotides used in this study. (DOCX) [file pone.0065168.s003.docx]

**Supplementary table S2: Oligonucleotides used in this study.**

T7 promoter sequences for generation of in vitro transcripts are written in bold letters. Restriction sites are underlined. 5’ Monophosphates are indicated (5’-).

| Oligonucleotide | Purpose | Plasmid | Sequence (5’ to 3’) |
| --- | --- | --- | --- |
| Pp_ibpA_probe_fw | Northern analysis; DNA-probe for *P. putida ibpA* | - | gaccgtttcaacgacctgtt |
| Pp_ibpA_probe_rv | Northern analysis; DNA-probe for *P. putida ibpA* | - | tcagttcagcgctggttttt |
| Pp_ibpA_RACE_rv | gene specific primer for 5’ RACE | - | AACGGAATGACGGAACAGTG |
| RACE_Adapterprimer | 5’ RACE PCR | - | GTCAGCAATCCCTAACGAG |
| RACE_Adapter | Adapter for 5’ RACE | - | GTCAGCAATCCCTAACGAG (GAG = Ribonucleotides) |
| Pp_ibpAprom_fw | *P. putida* *ibpA* with promoter region | pBO1033 | AAAGAATTCCACAGGCCGCCGTGTAGGAA |
| Pp_ibpA+150_fw | *P. putida* *ibpA* gene with 150 bp upstream | pBO1031 | atatgaattcgttcacccgcgaaga |
| Pp_ibpA+150_rv | *P. putida* *ibpA* gene with 150 bp downstream | pBO1031, pBO1033 | atataaagcttgccattaccgttacc |
| Pp_ibpA_PE_fw | Sequencing plasmid | pBO500 | cctacgcctgatcgaggat |
| Pp_ibpA_PE_rv | Sequencing plasmid; Primer extension | pBO500 | ggattcgaacaggtcgttga |
| Pp_runoff_fw | *P. putida ibpA* 5´UTR; runoff plasmid | pBO1513, pBO1514 | A**GAAATTAATACGACTCACTATAGGG**tacgcgatgccgaagccgggt |
| Pp_runoff_UTR_rv | *P. putida ibpA* 5´UTR and ΔhpI; runoff plasmid | pBO1513, pBO1569 | AGATATCAGTCATGGTCATAAACTCCTGAG |
| Pp_runoff_Toe_rv | *P. putida ibpA* 5´UTR + 60 bp coding; runoff plasmid | pBO1514 | AGATATCTCGTTGAAACGGTCGAAACCAA |
| Pa_runoff_fw | *P. aeruginosa ibpA* 5´UTR; runoff plasmid | pBO1515, pBO1516 | A**GAAATTAATACGACTCACTATAGGG**tgcgggatgccgaagacgggt |
| Pa_runoff_UTR_rv | *P. aeruginosa ibpA* 5´UTR and ΔhpI; runoff plasmid | pBO1515, pBO1571 | AAGCGCTCATTGCGAATCTCCTGAATTATC |
| Pa_runoff_Toe_rv | *P. aeruginosa ibpA* 5´UTR + 60 bp coding; runoff plasmid | pBO1516 | AGATATCGTTGAAGCGATCGAAGCCTA |
| Pp_ΔhpI_runoff_fw | *P. putida ibpA* 5´UTR ΔhpI; runoff plasmid | pBO1569 | A**GAAATTAATACGACTCACTATAGGG**atgacttacttgctgaatctc |
| Pa_ΔhpI_runoff_fw | *P. aeruginosa ibpA* 5´UTR ΔhpI; runoff plasmid | pBO1571 | A**GAAATTAATACGACTCACTATAGGG**tcagagtacttgctgatAAT |
| Pp_ibpA_fw | *P. putida ibpA* 5´UTR; pBAD-*bgaB* | pBO504 | aaagctagctacgcgatgccgaagccgggt |
| Pp_ibpA_rv | *P. putida ibpA* 5´UTR; pBAD-*bgaB* | pBO504 | aaagaattcagtcatggtcataaactcctgaga |
| Pp_ΔG39_fw | *P. putida ibpA* 5´UTR ΔG39 | pBO1040 | GCGTAATGACTTACTTCTGAATCTCAGGAGTT |
| Pp_ΔG39_rv | *P. putida ibpA* 5´UTR ΔG39 | pBO1040 | AACTCCTGAGATTCAGAAGTAAGTCATTACGC |
| Pp_U38C_fw | *P. putida ibpA* 5´UTR U38C | pBO2982 | GGTCGCGTAATGACTTACTCGCTGAATCTCAGGAGTTTA |
| Pp_U38C_rv | *P. putida ibpA* 5´UTR U38C | pBO2982 | TAAACTCCTGAGATTCAGCGAGTAAGTCATTACGCGACC |
| Pp_U38C/ΔG39_fw | *P. putida ibpA* 5´UTR U38C/ΔG39 | pBO2976 | cgcgtaatgacttactcctgaatctcagga |
| Pp_U38C/ΔG39_rv | *P. putida ibpA* 5´UTR U38C/ΔG39 | pBO2976 | taaactcctgagattcaggagtaagtcattacg |
| Pp_C36G_fw | *P. putida ibpA* 5´UTR C36G | pBO1044 | GGTCGCGTAATGACTTAGTTGCTGAATCTCAGGAG |
| Pp_C36G_rv | *P. putida ibpA* 5´UTR C36G | pBO1044 | CTCCTGAGATTCAGCAACTAAGTCATTACGCGACC |
| Pp_C40A_fw | *P. putida ibpA* 5´UTR C40A | pBO2983 | TCGCGTAATGACTTACTTGATGAATCTCAGGAGTTTATG |
| Pp_C40A_rv | *P. putida ibpA* 5´UTR C40A | pBO2983 | CATAAACTCCTGAGATTCATCAAGTAAGTCATTACGCGA |
| Pp_G42C_fw | *P. putida ibpA* 5´UTR G42C | pBO2984 | GCGTAATGACTTACTTGCTCAATCTCAGGAGTTTATGAC |
| Pp_G42C_rv | *P. putida ibpA* 5´UTR G42C | pBO2984 | GTCATAAACTCCTGAGATTGAGCAAGTAAGTCATTACGC |
| Pp_22/25A_fw | *P. putida ibpA* 5´UTR CGCG22-25AAAA | pBO2977 | atgccgaagccgggtAAAAtaatgacttactt |
| Pp_22/25A_rv | *P. putida ibpA* 5´UTR CGCG22-25AAAA | pBO2977 | GCAAGTAAGTCATTATTTTACCCGGCTTCGGC |

**Supplementary table S2 (part 2)**

| Oligonucleotide | Purpose | Plasmid | Sequence (5’ to 3’) |
| --- | --- | --- | --- |
| Pp_ΔhpI_fw | *P. putida ibpA* 5´UTR ΔhpI | pBO1566 | AAGCTAGCatgacttacttgctgaatctcaggagtttatgaccATGACTGAATTCA |
| Pp_ΔhpI_rv | *P. putida ibpA* 5´UTR ΔhpI | pBO1566 | TGAATTCAGTCATGGTCATAAACTCCTGAGATTCAGCAAGTAAGTCATGCTAGCTT |
| Pa_ibpA_fw | *P. aeruginosa ibpA* 5´UTR; pBAD-*bgaB* | pBO1046 | gctagctgcgggatgccga |
| Pa_ibpA_rv | *P. aeruginosa ibpA* 5´UTR; pBAD-*bgaB* | pBO1046 | GAATTCGCTCATTGCGAATCTC |
| Pa_ΔG39_fw | *P. aeruginosa ibpA* 5´UTR ΔG39 | pBO1505 | cgcatcagagtacttctgataattcaggag |
| Pa_ΔG39_rv | *P. aeruginosa ibpA* 5´UTR ΔG39 | pBO1505 | ctgaattatcagaagtactctgatgcggaa |
| Pa_ΔA35_fw | *P. aeruginosa ibpA* 5´UTR ΔA35 | pBO2979 | ttccgcatcagagtcttgctgataattcag |
| Pa_ΔA35_rv | *P. aeruginosa ibpA* 5´UTR ΔA35 | pBO2979 | aattatcagcaagactctgatgcggaaccc |
| Pa_C36A_fw | *P. aeruginosa ibpA* 5´UTR C36A | pBO1508 | ttccgcaccagagtaattgctgataattcag |
| Pa_C36A_rv | *P. aeruginosa ibpA* 5´UTR C36A | pBO1508 | aattatcagcaattactctggtgcggaaccc |
| Pa_C40A_fw | *P. aeruginosa ibpA* 5´UTR C40A | pBO1507 | cgcaccagagtacttgatgataattcaggag |
| Pa_C40A_rv | *P. aeruginosa ibpA* 5´UTR C40A | pBO1507 | ctgaattatcatcaagtactctggtgcggaa |
| Pa_23/26A_fw | *P. aeruginosa ibpA* 5´UTR CCGC23-26AAAA | pBO2987 | tgccgaagacgggttAAAAatcagagtacttg |
| Pa_23/26A_rv | *P. aeruginosa ibpA* 5´UTR CCGC23-26AAAA | pBO2987 | AGCAAGTACTCTGATTTTTAACCCGTCTTCGG |
| Pa_ΔhpI_fw | *P. aeruginosa ibpA* 5´UTR hairpin I deletion | pBO1565 | AAGCTAGCtcagagtacttgctgataattcaggagattcgcaatgAGCGAATTCA |
| Pa_ΔhpI_rv | *P. aeruginosa ibpA* 5´UTR hairpin I deletion | pBO1565 | TGAATTCGCTCATTGCGAATCTCCTGAATTATCAGCAAGTACTCTGAGCTAGCT |
| Psyr_*ibpA*_fw | *P. syringae* *ibpA* 5´UTR; pBAD-*bgaB* | pBO2954 | aaagctagcCAAGCGATGCCGAATACGGGT |
| Psyr_*ibpA*_rv | *P. syringae* *ibpA* 5´UTR; pBAD-*bgaB* | pBO2954 | aaagaattcCGCCATGATAATTTCTCCTGTTTG |
| Pstu_*ibpA*_fw | *P. stutzeri* *ibpA* 5´UTR; pBAD-*bgaB* | pBO2955 | aaagctagcAGCGGGATGCCGAAGTCG |
| Pstu_*ibpA*_rv | *P. stutzeri* *ibpA* 5´UTR; pBAD-*bgaB* | pBO2955 | aaagaattcACTCATGATAAACCTCCTGAAATC |
| Pmen_*ibpA*_fw | *P. mendocina* *ibpA* 5´UTR; pBAD-*bgaB* | pBO2956 | aaagctagcTGCGGGATGCCGAAGTCG |
| Pmen_*ibpA*_rv | *P. mendocina* *ibpA* 5´UTR; pBAD-*bgaB* | pBO2956 | aaagaattcGCTCATGAGTATTCTCCTGAAAAT |
