# Supplementary material for: Short ROSE-Like RNA Thermometers Control IbpA Synthesis in Pseudomonas Species
Source: PLoS One. 2013 May 31;8(5):e65168. doi: 10.1371/journal.pone.0065168 (PMC3669281; doi:10.1371/journal.pone.0065168)
Supplement: Table S3 — Plasmids used in this study. (DOCX) [file pone.0065168.s004.docx]

**Supplementary table S3: Plasmids used in this study**

Restriction sites used for cloning are given in brackets.

| Plasmid | Relevant Characteristics | Reference or source |
| --- | --- | --- |
| pUC18 | Cloning vector; Ap^r^ | [[1](#_ENREF_1)] |
| pBSL14 | Donor plasmids for [Km^r^]; Ap^r^ | [[2](#_ENREF_2)] |
| pEX18Tc | Suicide vector usable for *P. putida*; Tc^r^ | [[3](#_ENREF_3)] |
| pBAD-*bgaB* | pBO605; plasmid for translational *bgaB* reporter gene fusions with arabinose inducible promoter; Ap^r^ | [[4](#_ENREF_4)] |
| pBO500 | pUC18; 220 bp upstream to 80 bp coding region of the *P. putida ibpA* gene (SmaI) | This study |
| pBO1033 | pUC18; *P. putida* *ibpA* gene with promotor region (180 bp upstream and 150 bp downstream) (EcoRI/HindIII) | This study |
| pBO1031 | pUC18; *P. putida ibpA gene* with 150 bp upstream and downstream (EcoRI/HindIII) | This study |
| pBO1032 | pUC18; *P. putida ibpA* +/- 150 bp coding region and 32 bp deletion (PstI) of the *ibpA* gene (pBO1031) | This study |
| pBO1034 | pEX18Tc; *P. putida ibpA* +/- 150 bp coding region with 32 bp deletion (PstI) of the *ibpA* gene *(pBO1032)* | This study |
| pBO1037 | pEX18Tc; *P. putida ibpA* gene +- 150 bp with 32 bp deletion and Km cassette isertion [>Km^R^] into PstI site | This study |
| pBO1513 | pUC18; *P. putida ibpA* 5’ UTR with T7 promoter (SmaI) | This study |
| pBO1562 | pUC18; *P. putida ibpA* 5’ UTR ΔG_39_/U_38_C with T7 promoter | This study |
| pBO1569 | pUC18; *P. putida* *ibpA* 5’ UTR hairpin I deletion with T7 promoter (SmaI) | This study |
| pBO1514 | pUC18; *P. putida ibpA* 5’ UTR plus 60 nt coding region with T7 promoter (SmaI) | This study |
| pBO1564 | pUC18; *P. putida ibpA* 5’ UTR ΔG_39_/U_38_C plus 60 nt coding region with T7 promoter | This study |
| pBO1515 | pUC18; *P. aeruginosa* *ibpA* 5’ UTR with T7 promoter (SmaI) | This study |
| pBO1571 | pUC18; *P. aeruginosa* *ibpA* 5’ UTR hairpin I deletion with T7 promoter (SmaI) | This study |
| pBO1556 | pUC18; *P. aeruginosa* *ibpA* 5’ UTR ΔG_39_ with T7 promoter | This study |
| pBO1557 | pUC18; *P. aeruginosa* *ibpA* 5’ UTR ΔA_35_ with T7 promoter | This study |
| pBO1558 | pUC18; *P. aeruginosa* *ibpA* 5’ UTR GCCC_23-26_AAAA with T7 promoter | This study |
| pBO1516 | pUC18; *P. aeruginosa* *ibpA* 5’ UTR plus 60 nt coding region with T7-promoter (SmaI) | This study |
| pBO1559 | pUC18; *P. aeruginosa* *ibpA* 5’ UTR ΔG_39_ plus 60 nt coding region with T7-promoter | This study |
| pBO1560 | pUC18; *P. aeruginosa* *ibpA* 5’ UTR ΔA_35_ plus 60 nt coding region with T7-promoter | This study |
| pBO1561 | pUC18; *P. aeruginosa* *ibpA* 5’ UTR GCCC_23-26_AAAA plus 60 nt coding region with T7-promoter | This study |
| pBO417 | pBAD-*bgaB*; *E. coli ibpA* 5’ UTR (NheI/EcoRI) | [[5](#_ENREF_5)] |
| pBO504 | pUC18; *P. putida ibpA* 5’ UTR (SmaI) | This study |
| pBO1039 | pBAD-*bgaB*; *P. putida ibpA* 5’ UTR (NheI/EcoRI) | This study |
| pBO1040 | pBAD-*bgaB*; *P. putida ibpA* 5’ UTR ΔG_39_ (NheI/EcoRI) | This study |
| pBO2982 | pBAD-*bgaB*; *P. putida ibpA* 5’ UTR U_38_C (NheI/EcoRI) | This study |
| pBO2976 | pBAD-*bgaB*; *P. putida ibpA* 5’ UTR U_38_C/ΔG_39_ (NheI/EcoRI) | This study |
| pBO1044 | pBAD-*bgaB*; *P. putida ibpA* 5’ UTR C_36_G (NheI/EcoRI) | This study |
| pBO2983 | pBAD-*bgaB*; *P. putida ibpA* 5’ UTR C_40_A (NheI/EcoRI) | This study |
| pBO2984 | pBAD-bgaB; *P. putida ibpA* 5’ UTR G_42_C (NheI/EcoRI) | This study |
| pBO2977 | pBAD-*bgaB*; *P. putida ibpA* 5’ UTR CGCG_22_25_AAAA (NheI/EcoRI) | This study |
| pBO1566 | pUC18; *P. putida ibpA* 5’ UTR hairpin I deletion (SmaI) | This study |
| pBO2978 | pBAD-*bgaB*; *P. putida ibpA* 5’ UTR hairpin I deletion (NheI/EcoRI) | This study |

**Supplementary table S3 (part 2)**

| Plasmid | Relevant Characteristics | Reference or source |
| --- | --- | --- |
| pBO1046 | pUC18; *P. aeruginosa ibpA* 5’ UTR (HincII) | This study |
| pBO1047 | pBAD-*bgaB*; *P. aeruginosa ibpA* 5’ UTR (NheI/EcoRI) | This study |
| pBO1505 | pBAD-*bgaB*; *P. aeruginosa ibpA* 5’ UTR ΔG_39_ (NheI/EcoRI) | This study |
| pBO2979 | pBAD-*bgaB*; *P. aeruginosa ibpA* 5’ UTR ΔA_35_ (NheI/EcoRI) | This study |
| pBO1508 | pBAD-*bgaB*; *P. aeruginosa ibpA* 5’ UTR C_36_A (NheI/EcoRI) | This study |
| pBO1507 | pBAD-*bgaB*; *P. aeruginosa ibpA* 5’ UTR C_40_A (NheI/EcoRI) | This study |
| pBO2987 | pBAD-*bgaB*; *P. aeruginosa ibpA* 5’ UTR CCGC_23-26_AAAA (NheI/EcoRI) | This study |
| pBO1565 | pUC18; *P. aeruginosa ibpA* 5’ UTR hairpin I deletion (SmaI) | This study |
| pBO2985 | pBAD-*bgaB*; *P. aeruginosa ibpA* 5’ UTR hairpin I deletion (NheI/EcoRI) | This study |
| pBO2954 | pUC18; *P. syringae* *ibpA* 5’ UTR (SmaI) | This study |
| pBO2955 | pUC18; *P. stutzeri ibpA* 5’ UTR (SmaI) | This study |
| pBO2956 | pUC18; *P. mendocina ibpA* 5’ UTR (SmaI) | This study |
| pBO2968 | pBAD-*bgaB*; *P. syringae ibpA* 5’ UTR (NheI/EcoRI) | This study |
| pBO2969 | pBAD-*bgaB*; *P. stutzeri* *ibpA* 5’ UTR (NheI/EcoRI) | This study |
| pBO2967 | pBAD-*bgaB*; *P. mendocina* *ibpA* 5’ UTR (NheI/EcoRI) | This study |

**References**

1. Yanisch-Perron C, Vieira J, Messing J (1985) Improved M13 phage cloning vectors and host strains: nucleotide sequences of the M13mp18 and pUC19 vectors. Gene 33: 103-119.

2. Alexeyev MF (1995) Three kanamycin resistance gene cassettes with different polylinkers. Biotechniques 18: 52, 54, 56.

3. Hoang TT, Karkhoff-Schweizer RR, Kutchma AJ, Schweizer HP (1998) A broad-host-range Flp-FRT recombination system for site-specific excision of chromosomally-located DNA sequences: application for isolation of unmarked *Pseudomonas aeruginosa* mutants. Gene 212: 77-86.

4. Waldminghaus T, Gaubig LC, Narberhaus F (2007) Genome-wide bioinformatic prediction and experimental evaluation of potential RNA thermometers. Mol Genet Genomics 278: 555-564.

5. Waldminghaus T, Gaubig LC, Klinkert B, Narberhaus F (2009) The *Escherichia coli ibpA* thermometer is comprised of stable and unstable structural elements. RNA Biol 6: 455-463.
